# Association between insulin-like growth factor-1 receptor (IGF1R) negativity and poor prognosis in a cohort of women with primary breast cancer

**DOI:** 10.1186/1471-2407-14-794

**Published:** 2014-11-03

**Authors:** Kristina E Aaltonen, Ann H Rosendahl, Hans Olsson, Per Malmström, Linda Hartman, Mårten Fernö

**Affiliations:** Division of Oncology and Pathology, Department of Clinical Sciences Lund, Lund University, Medicon Village, SE-223 81 Lund, Sweden; Skåne Department of Oncology, Skåne University Hospital, Lund, Sweden; Department of Clinical Pathology and Clinical Genetics, Östergötland County Council, Linköping, Sweden; Department of Clinical and Experimental Medicine, Molecular and Immunological Pathology, Faculty of Health Sciences, Linköping University, Linköping, Sweden; Regional Cancer Centre South, Lund, Sweden

**Keywords:** Primary breast cancer, Insulin-like growth factor-1 receptor, Estrogen receptor, Tamoxifen, Prognosis

## Abstract

**Background:**

Resistance towards endocrine therapy is a great concern in breast cancer treatment and may partly be explained by the activation of compensatory signaling pathways. The aim of the present study was to investigate if the insulin-like growth factor-1 receptor (IGF1R) signaling pathway was activated or deregulated in breast cancer patients and to explore if any of the markers were prognostic, with or without adjuvant tamoxifen. This signaling pathway has been suggested to cause estrogen independent cell growth and thus contribute to resistance to endocrine treatment in estrogen receptor (ER) positive breast cancer.

**Methods:**

The protein expression of IGF1R, phosphorylated Mammalian Target of Rapamycin (p-mTOR) and phosphorylated S6 ribosomal protein (p-S6rp) were investigated by immunohistochemistry using tissue microarrays in two patient cohorts. Cohort I (N = 264) consisted of mainly postmenopausal women with stage II breast cancer treated with tamoxifen for 2 years irrespective of ER status. Cohort II (N = 206) consisted of mainly medically untreated, premenopausal patients with node-negative breast cancer. Distant disease-free survival (DDFS) at 5 years was used as end-point for survival analyses.

**Results:**

We found that lower IGF1R expression was associated with worse prognosis for tamoxifen treated, postmenopausal women (HR = 0.70, 95% CI = 0.52 – 0.94, p = 0.016). The effect was seen mainly in ER-negative patients where the prognostic effect was retained after adjustment for other prognostic markers (adjusted HR = 0.49, 95% CI = 0.29 – 0.82, p = 0.007). Expression of IGF1R was associated with ER positivity (p < 0.001) in the same patient cohort.

**Conclusions:**

Our results support previous studies indicating that IGF1R positivity reflects a well differentiated tumor with low metastatic capacity. An association between lack of IGF1R expression and worse prognosis was mainly seen in the ER-negative part of Cohort I. The lack of co-activation of downstream markers (p-mTOR and p-S6rp) in the IGF1R pathway suggested that the prognostic effect was not due to complete activation of this pathway. Thus, no evidence could be found for a compensatory function of IGF1R signaling in the investigated cohorts.

**Electronic supplementary material:**

The online version of this article (doi:10.1186/1471-2407-14-794) contains supplementary material, which is available to authorized users.

## Background

Breast cancer is a common disease in the Western world and one in eight women gets the diagnosis during her lifetime. Breast cancer treatment is often successful and therapy can be targeted based on the expression of biomarkers such as the estrogen receptor (ER) and human epidermal growth factor receptor 2 (HER2). However, approximately 50% of patients with ER-positive disease are resistant to ER directed therapy and of the ones that initially respond many will develop resistance during therapy [1]. As no single mechanism can explain all cases of resistance, the study of alternative/compensatory signaling pathways is important for future treatment combinations to decrease the risk of adaptive resistance. Expression of predictive biomarkers in addition to ER and HER2 at the initiation of therapy could also provide guidance to the choice of treatment.

**Figure 1:**
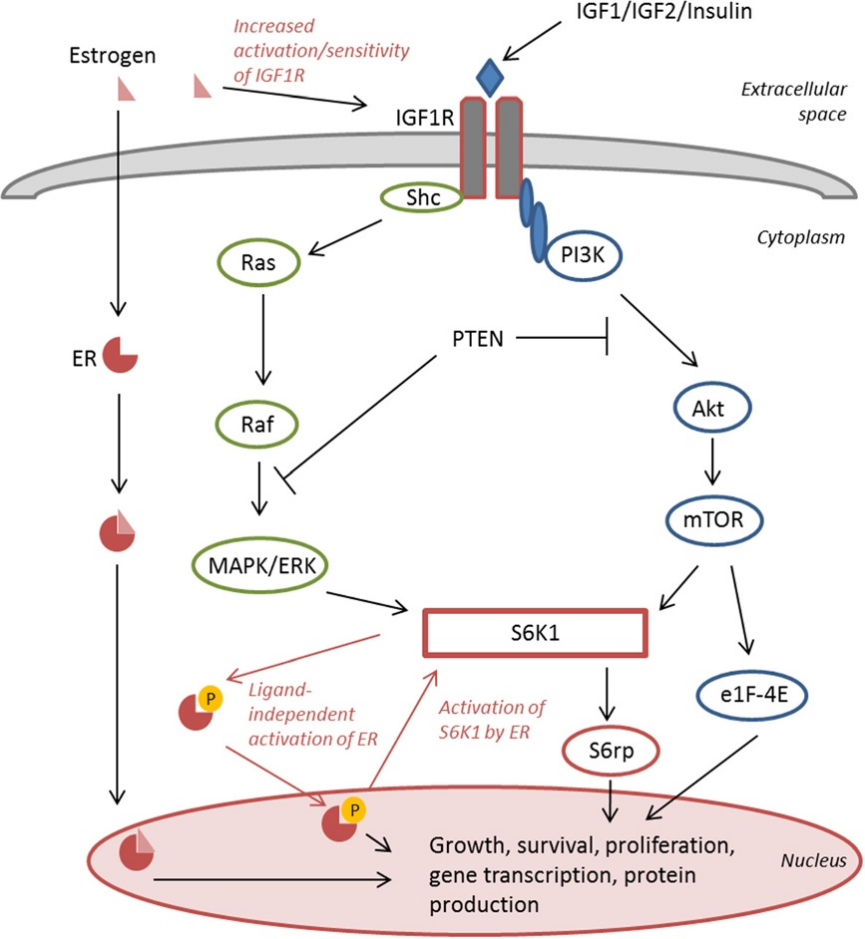
**Schematic illustration of the IGF1R/mTOR signaling pathway resulting in growth and survival of the cell.** Examples of cross-talk between the IGF1R signaling pathway and estrogen and the estrogen receptor (ER) are shown.

Activation of the insulin-like growth factor-1 receptor (IGF1R) is essential for survival of many oncogenic cells and its important role in cancer is well established [2]. In normal tissue, activation of IGF1R by its ligands IGF-I and IGF-II is important for regulation of cell differentiation, proliferation and metabolism and *IGF1R* gene transcription has been found to be suppressed by functional tumor suppressor genes such as *BRCA1* [3] and *p53* [4, 5]. It has also been shown that estrogens and ER can increase IGF1R signaling [6, 7] and IGF1R can in its turn phosphorylate ER through its downstream activator S6K1 leading to ligand-independent activation of ER [8] (Figure 1). This crosstalk between IGF1R and ER has led to the proposal of combined anti-IGF1R and anti-ER therapies to decrease resistance development in ER-positive breast cancer [9]. Downstream of IGF1R, activation of several substrates and phosphorylation events in the signaling cascade (Figure 1) also provides possibilities for combined treatment. Mammalian Target of Rapamycin (mTOR) is part of the common PI3K/Akt signaling pathway that transfers proliferative signals from a number of different receptor tyrosine kinases (RTKs), including IGF1R. Upon stimulation, mTOR induces activation of S6K1 with subsequent phosphorylation of S6 ribosomal protein (S6rp) resulting in an increase in mRNA translation and cell proliferation. S6K1 can also be activated by the Ras/MEK/MAPK-cascade, another possible pathway transferring growth promoting signals from IGF1R [10] (Figure 1).

*In vitro* experiments have shown promising results for targeting this pathway in combination with endocrine therapy [11, 12]. However, clinical studies have yet to prove a positive effect of IGF1R inhibition in the therapeutic setting and it is possible that selection of patients appropriate for this type of treatment is needed. Targeting mTOR together with endocrine therapy in metastatic breast cancer has provided successful results with prolonged progression-free survival in the large BOLERO-2 study [13] and improved clinical benefit rate, time to progression and overall survival in the GINECO study [14]. Combined therapy against mTOR and IGF1R is currently investigated in clinical trials [15].

Studies of the prognostic role of IGF1R in breast cancer have so far given discrepant results. A few studies have found that high expression of the IGF1R protein [16] or mRNA [17] was associated with shorter survival and worse prognosis, whereas other studies have found an association between longer survival and high IGF1R expression [18–21]. High levels of phosphorylation of mTOR or S6K1 are indicative of activation of several signaling pathways and not solely indicative for IGF1R activation. High mTOR expression has been associated with aggressive disease and higher risk of recurrence [22, 23] and phosphorylation of mTOR has also been found to increase with disease progression [24]. In a recent study, high p-mTOR expression was associated with decreased tamoxifen response [25]. S6K1 overexpression has been found in high-grade breast cancers [23] and when co-expressed with IGF1R it has been related to poor survival in all breast cancer subtypes [16].

The aim of this study was to investigate if IGF1R and its downstream pathway was activated or deregulated in primary breast cancer and to explore if any of the markers were prognostic, with or without adjuvant tamoxifen. We hypothesized that overexpression of IGF1R, possibly in combination with over-activation of the downstream markers mTOR and S6rp, could be associated with worse prognosis for ER-positive patients treated with tamoxifen. Two cohorts (one tamoxifen treated and one mainly without systemic treatment) were included in the study to investigate the predictive and prognostic value of marker expression. However, the results showed that *negative* IGF1R was associated with worse prognosis in one of the investigated cohorts and no indications of overactivation of the complete pathway could be found. IGF1R expression was positively associated with ER expression and our results suggest that high IGF1R expression is associated with well differentiated tumors with low metastatic capacity. Whenever applicable in the study, the REMARK recommendations for reporting of tumor marker studies were followed [26].

## Methods

### Patient cohorts

Cohort I consisted of mainly postmenopausal patients who were all treated with tamoxifen for 2 years irrespective of ER status. The original, prospective study included 445 patients diagnosed with stage II breast carcinoma in the South Swedish Health Care Region between 1985 and 1994, and has been described in detail previously [27–31]. In addition to tamoxifen, therapy consisted of either breast conserving surgery and postoperative radiotherapy or modified radical mastectomy in combination with radiotherapy (50 Gy) for patients with lymph node-positive cancer. 264 patients, of whom 55 (21%) were premenopausal and 209 (79%) were postmenopausal, could be evaluated in the present study. Two of the premenopausal patients received adjuvant chemotherapy in addition to tamoxifen. The median follow-up for distant disease-free survival (DDFS) was 6.1 years for patients free of distant metastases and alive at the latest review of the patients’ record.

Cohort II consisted of 237 premenopausal patients with lymph-node negative breast cancer identified in the South Swedish Breast Cancer Region between 1991 and 1994. The original prospective study has been described previously [32, 33]. All patients underwent radical surgery for early breast cancer and 117 of the patients received post-operative radiotherapy. 206 patients could be evaluated for IGF1R in the present study and 28 of these patients were given adjuvant therapy (19 received chemotherapy and 9 received endocrine therapy). Median follow-up for DDFS was 10.9 years for patients alive and free from distant metastases at the latest review of the patients’ records.

The original studies, as well as the present follow-up study, of the two cohorts were approved by the Ethics committee of Lund University.

### Tissue microarray and immunohistochemistry

Tissue microarrays (TMAs) were constructed from paraffin blocks of the primary tumors. Two core biopsies (1.0 mm in diameter) were punched out from representative areas of each invasive breast cancer and mounted into a recipient block using a manual TMA machine (Beecher Instruments, Sun Prairie, WI, USA). 3–4 μm sections of the recipient blocks were mounted on three separate slides and stained with three different antibodies using an automatic immunohistochemistry machine (Autostainer, DAKO, Glostrup, Denmark) according to standard procedures. Antigen retrieval for IGF1Rβ was done under pressure in Tris-EDTA buffer (pH = 6). The antibody (#3027, CellSignaling technology, Boston, MA, USA) was diluted 1:300 and incubated in room temperature for 1 hour. The antibodies phospho-mTOR (Ser2448, #2976, CellSignaling technology) and phospho-S6rp (Ser235/236, #4858, CellSignaling technology) were diluted 1:50 and 1:100, respectively. Antigen retrieval for p-mTOR and p-S6rp were done in Tris-EDTA buffer (pH = 9) and incubation was performed for 30 minutes in room temperature. Breast cancer cases with strong positive staining as well as completely negative staining could be identified with the dilutions stated above.

### Biomarker evaluation

**Figure 2:**
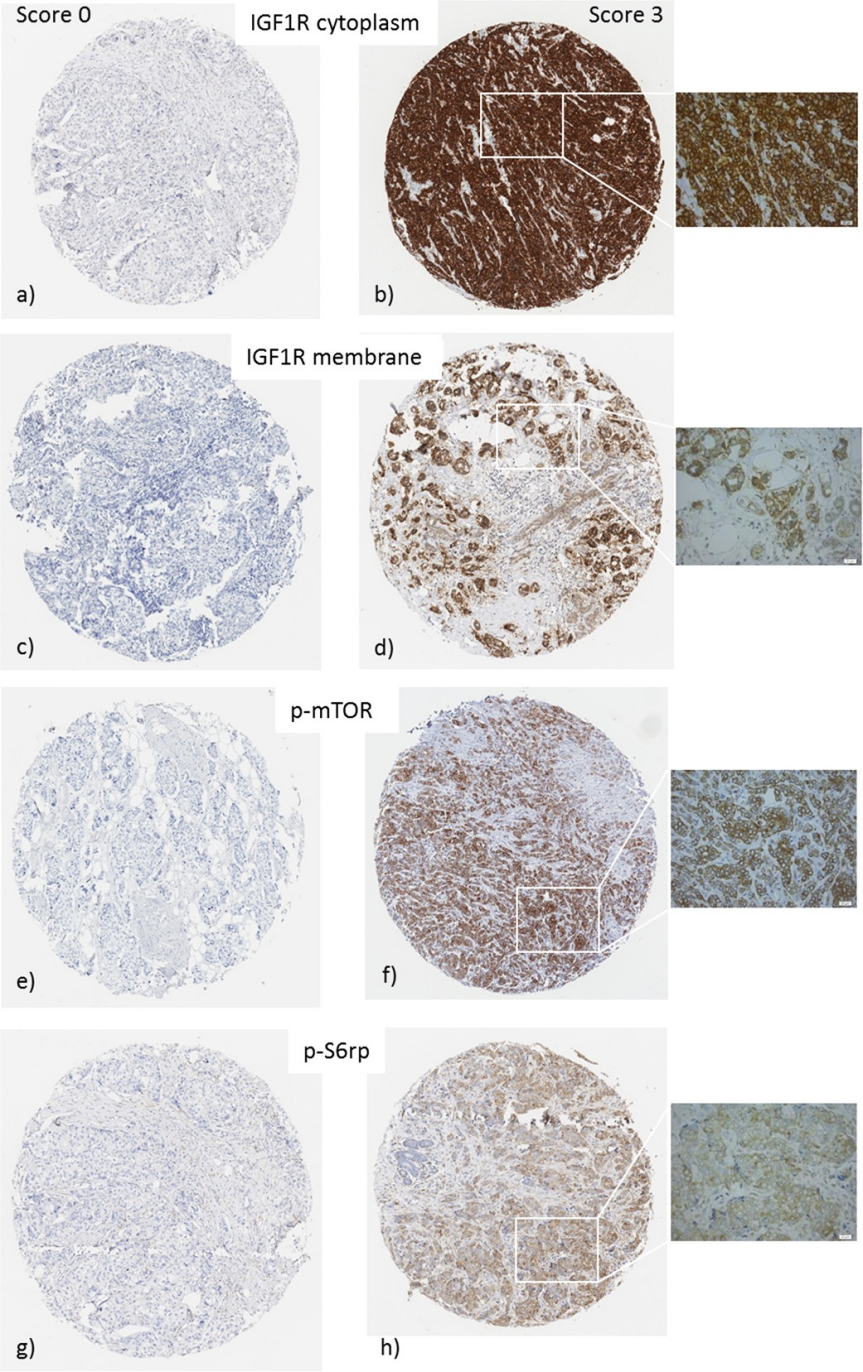
**Staining of experimental markers.** IGF1R cytoplasm **(a and b)**, IGF1R membrane **(c and d)**, p-mTOR **(e and f)** and p-S6rp **(g and h)**. Pictures on the left **(a, c, e and g)** show score 0 (negative) and pictures on the right show score 3 (strong). Pictures by LRI (Lund, Sweden). Original magnification 10x (TMA cores) and 40x (insert).

All slides (IGF1R, p-mTOR and p-S6rp) were digitized with the ScanScope XT (Aperio, Vista, CA) by LRI Instruments (Lund, Sweden) and were evaluated by two independent scorers (HO and KA). Cytoplasmic staining was evaluated for all three antibodies and the fraction of stained cancer cells was scored as 0, 1, 5, 10, 20, 30, 40, 50, 60, 70, 80, 90, 95, 99%. The cytoplasmic staining intensity was evaluated as negative (0), weak (1), moderate (2) or strong (3). For IGF1R, membrane staining was evaluated by a system adapted from HER2 staining criteria implemented by Hercep Test™ (DAKO) and scored as 0 (negative), 1 (weak and incomplete membrane staining), 2 (weak, circumferential staining in more than 10% of cells) or 3 (uniform, intense circumferential staining in more than 10% of the cells). TMA cores with only cancer *in situ* or with less than 100 cancer cells were considered non-evaluable. The highest result of the two core biopsies was selected if antigen expression was heterogeneous. Discordant cases were re-examined and a consensus decision was made. Examples of typical staining with experimental markers are shown in Figure 2. Evaluation of tumor characteristics and standard markers was done as previously described for Cohort I [27–31] and Cohort II [32, 33]. Both cohorts were subdivided into four different subgroups based on St Gallen criteria [34]: Luminal A-like (ER+, PgR+, Ki67 low, HER2-), Luminal B-like (ER + and PgR- and/or Ki67 high and/or HER2+), Triple-negative (ER-, PgR-, HER2-), and HER2-positive (ER-, PgR-, HER2+). However, expression of ER and the progesterone receptor (PgR) were evaluated with cytosol enzyme immunoassay as previously described [29] and the cut-off for positivity was necessarily different from the latest St Gallen recommendations [34].

### Statistical analyses

Association between the expression of IGF1R, p-mTOR and p-S6rp and other prognostic factors was evaluated using Mann–Whitney-test (binary variables) and Spearman’s rank correlation (continuous variables). In Cohort I, 10 patients could not be included in any St Gallen subgroup due to PgR positivity and ER negativity and these patients were excluded from subgroup analyses. In Cohort II, 19 patients were excluded from the analyses for the same reason. A stability test including these patients in the Luminal A or Luminal B-like subgroup (depending on HER2 and Ki67 expression) did not give divergent results. Differences in the distribution of experimental markers between subgroups were investigated with Kruskal-Wallis equality-of-populations rank test corrected for ties, follo-wed by pairwise Mann–Whitney tests, which are reported uncorrected for multiple testing.

DDFS with 5 year follow-up was used as endpoint in prognostic analyses of the experimental markers. DDFS was estimated and plotted using the Kaplan-Meier method, and the log-rank test for trend was used to evaluate the effect of the investigated factors on survival. Cox proportional hazard regression was used in univariable analyses to obtain hazard ratios (HR), and for multivariable analyses including interaction testing. In multivariable analyses, tu-mor size, node status (only Cohort I), ER expression, Ki67 expression, HER2 status, and menopausal status (Cohort I) or age (Cohort II), were included. The two cohorts were independently analyzed and both materials were also subdivided into ER-positive and ER-negative patients. Separate survival analyses including only postmenopausal (N = 209) and only node-positive patients (N = 178), respectively, were done in Cohort I. Hazard ratio differences between strata were compared by testing for interaction in the Cox-model. In Cohort II, survival analyses were repeated without the 28 patients that had received adjuvant endocrine or chemotherapeutic treatment.

For IGF1R expression, 97% of the non-negative tumors were classified as 95% - 99% positive cells and thus, no additional information would be provided by including the fraction of stained cells into the analyses. Thus, the reported results are based on the intensity of staining only. p-mTOR and p-S6rp staining were more variable regarding fraction and an H-score system (intensity x fraction resulting in four groups with scores 0–10, 11–100, 101–200 and 201–300) was evaluated for analysis of these markers. However, limited additional information was obtained by including fraction into the analyses and the presented results are based on intensity scoring only if nothing else is stated.

All statistical calculations were done in STATA (StataCorp/SE 11.2 for Windows. 2011. College Station, TX, USA).

## Results

### Association between tumor characteristics and IGF1R, p-mTOR and p-S6rp

**Figure 3:**
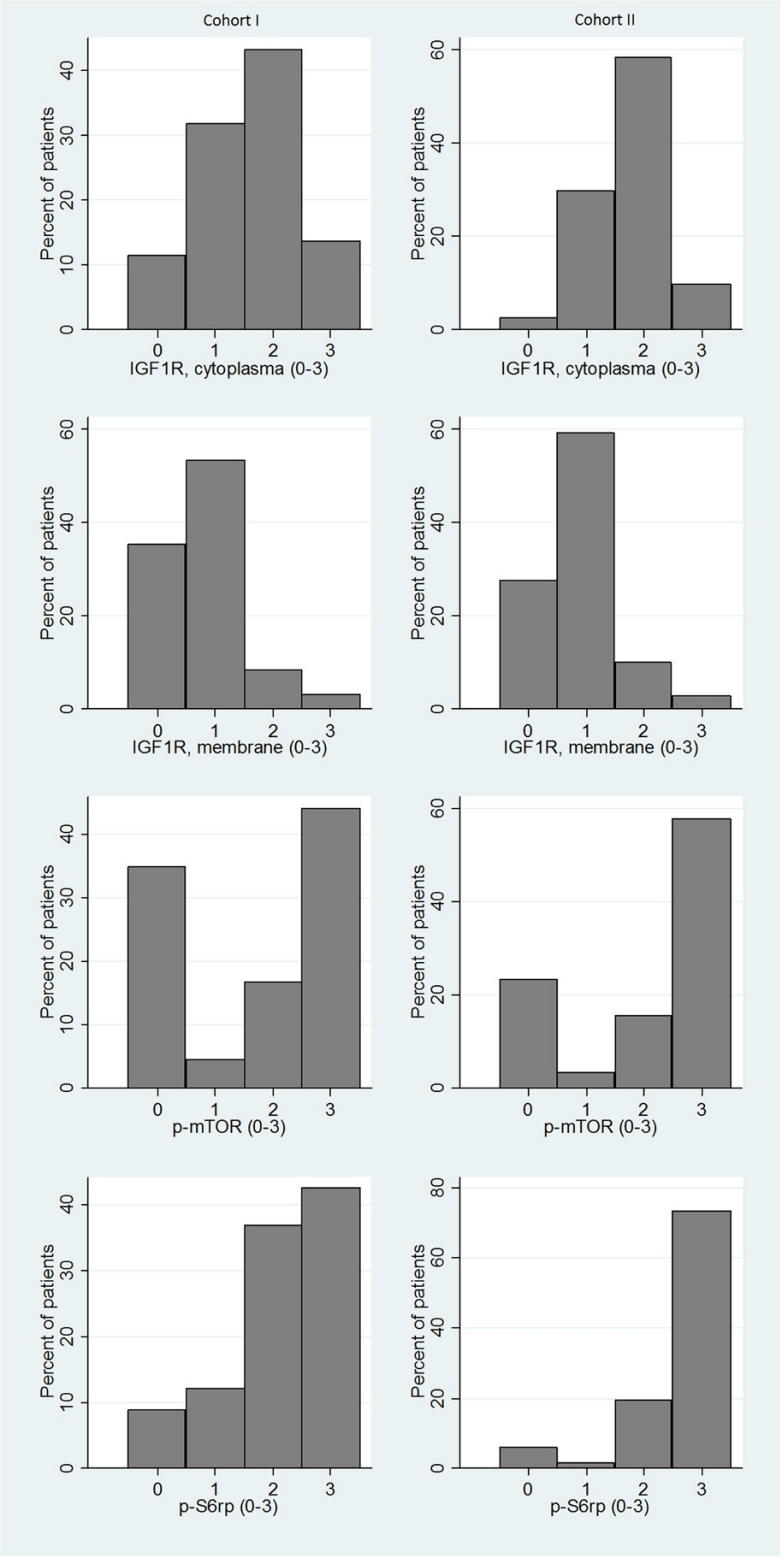
**Distribution of staining intensities for the experimental markers.**

The distribution of staining intensities for the different experimental markers is illustrated in Figure 3. For both cohorts, the association between IGF1R cytoplasm intensity and tumor characteristics can be found in Table 1. Data from IGF1R membrane staining gave comparable results and can be found in detail in Additional file 1 together with data from p-mTOR and IGF1R staining. Notable is that in Cohort I there was very strong evidence of a positive association between ER/PgR positivity and a high expression of IGF1R (p < 0.001). High p-S6rp was strongly associated with hormone receptor positivity (p < 0.001 for both ER and PgR). For p-mTOR there was very strong evidence for a positive association with Ki67 expression (p < 0.001), and slight evidence for an association with ER positivity (p = 0.068). In Cohort II, high p-mTOR expression was associated with ER positivity (p = 0.014) and higher age (p = 0.026), whereas it was negatively associated with Ki67 expression (p = 0.010). p-S6rp expression was positively associated with Ki67 expression and histological grade (both p < 0.001), and negatively associated with ER and PgR expression (both p < 0.001). See Table 1 for IGF1R cytoplasmic expression and Additional file 1 for IGF1R membrane expression, p-mTOR and p-S6rp expression.

**Table 1:** **Cytoplasmic intensity of IGF1R expression in relation to tumor and patient characteristics for Cohort I (N = 264) and Cohort II (N = 206)**

	Cohort I	% of patients with different expression levels					Cohort II	% of patients with different expression levels				
	N	Neg	Weak	Moderate	Strong	p-value	N	Neg	Weak	Moderate	Strong	p-value
**Total**	264	11	32	43	14		206	2	30	58	10	
**Age**												
Median age	264	63^a^	61^a^	63^a^	60^a^	0.34^b^	206	45^a^	46^a^	47^a^	47^a^	0.026^b^
**Menopausal status**												
Pre	55	7	29	45	18	0.15^c^	n/a					
Post	209	12	33	43	12							
**Tumor size**												
0 – 20 mm	78	8	35	50	8	0.83^c^	156	3	30	58	10	0.74^c^
>20 mm	186	13	31	40	16		50	2	28	60	10	
**Node status**												
N0	86	12	35	41	13	0.52^c^	n/a					
N+	178	11	30	44	14							
**NHG**												
1 – 2	186	8	32	45	15	0.086^c^	138	1	29	62	9	0.39^c^
3	75	19	31	39	12		66	6	32	50	12	
Missing	3						2					
**ER**												
Positive	174	3	29	49	18	<0.001^c^	139	1	27	63	9	0.32^c^
Negative	80	29	38	29	5		67	4	34	49	12	
Missing	10						0					
**PgR**												
Positive	133	2	29	52	17	<0.001^c^	149	1	28	63	7	0.66^c^
Negative	121	21	34	33	12		57	5	33	46	16	
Missing	10						0					
**Ki67**												
Low	162	8	33	43	16	0.095^c^	125	2	32	59	7	0.33^c^
High	99	17	29	43	10		61	3	28	52	16	
Missing	3						20					
**HER2**												
Negative	199	9	32	44	15	0.042^c^	171	1	29	60	9	0.027^c^
Positive	33	21	33	39	6		22	14	41	36	9	
Missing	32						13					
**St Gallen subgroups** ^**d**^												
Luminal A-like	72	4	32	46	18	<0.001^e^	92	0	27	64	9	0.24^e^
Luminal B-like	80	4	26	51	19		32	3	31	53	13	
Triple-negative	42	26	43	26	5		32	3	41	41	16	
HER2+ (non-luminal)	18	39	33	22	6		8	25	38	25	13	
Missing	52						42					

Abbreviations: *ER* = Estrogen receptor, *PgR* = Progesterone receptor, *HER2* = Human epidermal growth factor receptor 2, *NHG* = Histological grade according to Elston and Ellis [35], *n/a* = Not applicable.

^a^Median age in the different groups.

^b^Spearman’s rank-correlation.

^c^Mann–Whitney test.

^d^See (34) for complete definition of St Gallen subgroups.

^e^Kruskal-Wallis test.

Between the experimental markers, strong positive association was found between IGF1R expression in cytoplasm and IGF1R expression in the membrane in both cohorts (p < 0.001). In Cohort I, moderate evidence for positive association between IGF1R cytoplasmic staining and p-mTOR staining could also be found (p = 0.038).

### Expression of experimental markers in St Gallen subgroups

In Cohort I, the subgroups defined in St Gallen International Guidelines [34] differed in the expression of IGF1R (p < 0.001 for both cytoplasmic and membrane staining; Table 1 and Additional file 1). Pairwise comparisons revealed that IGF1R intensity was higher in Luminal A-like (N = 72) and Luminal B-like (N = 80) subgroups compared to the Triple-negative (N = 42) and HER2-positive (N = 18) subgroups (all p < 0.001 for both cytoplasmic and membrane staining). However, no difference in expression was found between the Luminal A and B-like groups or between the Triple-negative and HER2-positive groups (p > 0.4 for all comparisons). Lack of p-mTOR expression was most common in the Triple-negative subgroup compared to the other subgroups (p < 0.04). No difference in expression of p-S6rp could be found. In Cohort II, no difference in IGF1R intensity or p-S6rp intensity could be found between St Gallen subgroups. Expression of p-mTOR was higher in the Luminal subgroups compared to Triple-negative (both comparisons p < 0.001) and also higher in HER2-positive compared to the Triple-negative subgroup (p = 0.010). The group sizes were 92 patients in Luminal A-like, 32 in Luminal B-like, 32 in Triple-negative and 8 in HER2-positive.

### Prognostic value of the experimental markers

**Figure 4:**
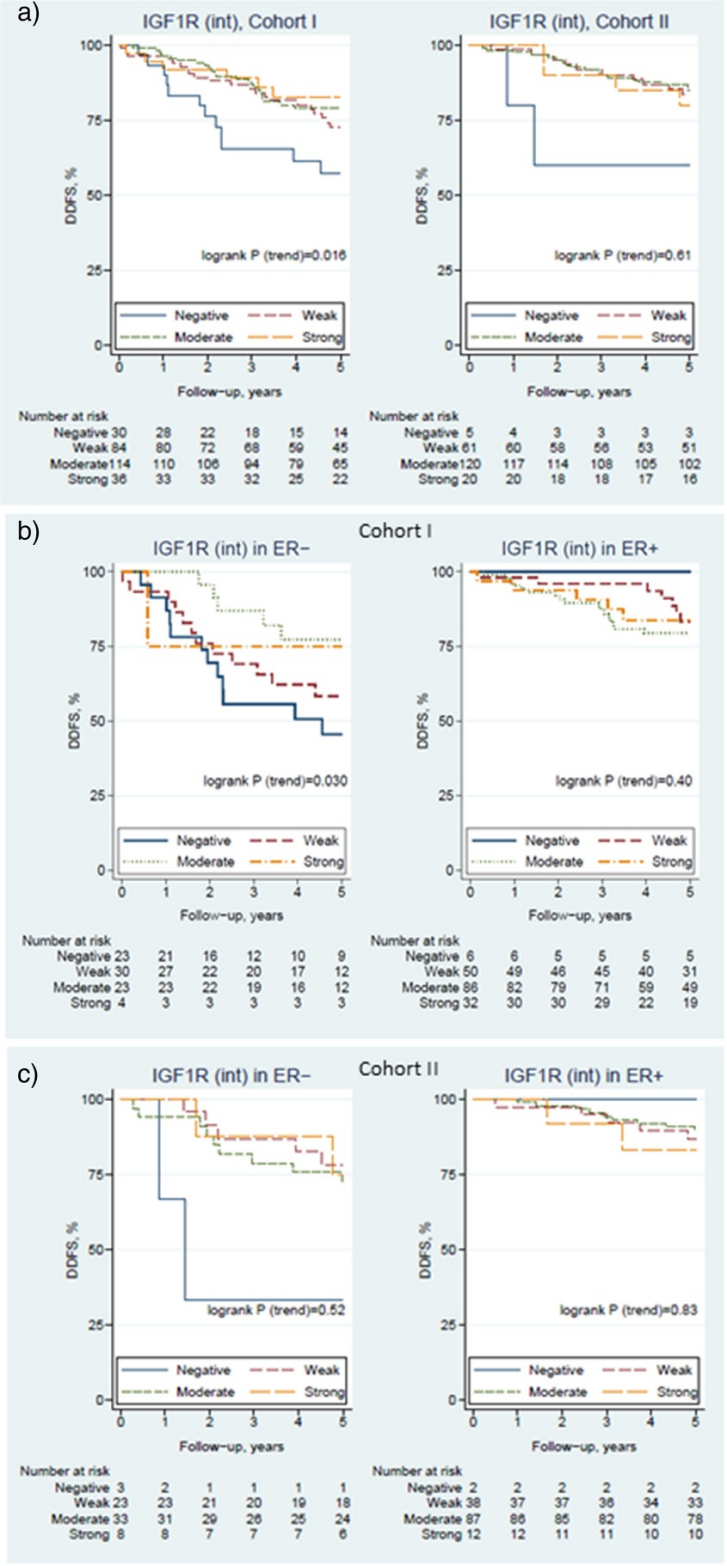
**Distant disease-free survival (DDFS) for patients based on expression of IGF1R in the cytoplasm.** The Kaplan-Meier curves show **a)** all patients in Cohort I (N = 264) and Cohort II (N = 206), and patients stratified on ER status for **b)** Cohort I and **c)** Cohort II.

In the tamoxifen treated Cohort I, Kaplan-Meier analysis for IGF1R showed worse prognosis for patients lacking IGF1R expression (Figure 4a). Cytoplasmic and membrane staining gave comparable results in all analyses, and only results from the cytoplasmic staining of IGF1R are presented in the text (see Tables 2 and 3 for membrane expression). Cox-regression gave a Hazard ratio (HR) of 0.70 per intensity step (95% CI = 0.52 – 0.94, p = 0.016, Table 2), but the prognostic value of IGF1R cytoplasmic expression was not retained in multivariable analyses among all patients in Cohort I (Table 2a).

**Table 2:** **Prognostic value of IGF1R cytoplasm intensity in Cohort I (a) and II (b)**

a)								
Cohort I	DDFS univariable				DDFS multivariable ^b^			
Variable	N	HR	95% CI	p-value ^a^	N	HR	95% CI	p-value
IGF1R cytoplasm (0–3, linear)	264	0.70	0.52-0.94	0.016	220	0.80	0.58-1.1	0.18
Node status (N + vs N0)	264	1.2	0.71-2.1	0.45	220	1.1	0.57-2.0	0.83
Tumor size (>20 mm vs ≤20 mm)	264	2.0	1.0-3.8	0.037	220	1.7	0.80-3.5	0.17
HER2 (pos vs neg)	232	2.0	1.0-3.8	0.037	220	1.3	0.69-2.7	0.39
ER (pos vs neg)	254	0.38	0.23-0.64	<0.001	220	0.56	0.31-1.0	0.062
Ki67 (>20% vs ≤20%)	261	2.4	1.4-3.9	0.001	220	1.5	0.79-2.7	0.23
Menopausal status (post vs pre)	264	0.32	0.19-0.53	<0.001	220	0.37	0.20-0.68	0.001
IGF1R membrane (0–3, linear)	264	0.58	0.39-0.86	0.007				
p-mTOR (0–3, linear)	264	0.94	0.78-1.1	0.54				
p-S6rp (0–3, linear)	264	0.97	0.75-1.3	0.84				
Age (years, linear)	264	0.97	0.94-0.99	0.003				
Histologic grade (3 vs 1–2)	261	2.2	1.3-3.6	0.003				
PgR (pos vs neg)	254	0.61	0.37-1.0	0.064				
**b)**								
**Cohort II**	**DDFS univariable**				**DDFS multivariable** ^**c**^			
**Variable**	**N**	**HR**	**95% CI**	**p-value** ^**a**^	**N**	**HR**	**95% CI**	**p-value**
IGF1R cytoplasm (0–3, linear)	206	0.87	0.52-1.5	0.61	179	1.0	0.59-1.8	0.91
Age (years, linear)	206	0.91	0.86-0.96	0.001	179	0.92	0.86-0.99	0.02
Tumor size (>20 mm vs ≤20 mm)	206	1.9	0.94-3.8	0.07	179	1.2	0.51-2.7	0.70
HER2 (pos vs neg)	193	6.0	2.9-13	<0.001	179	5.1	2.3-11	<0.001
ER (pos vs neg)	206	0.38	0.20-0.75	0.005	179	0.86	0.38-1.9	0.71
Ki67 (>20% vs ≤20%)	186	2.6	1.3-5.2	0.007	179	1.8	0.75-4.1	0.19
IGF1R membrane (0–3, linear)	206	0.87	0.53-1.4	0.57				
p-mTOR (0–3, linear)	205	0.94	0.72-1.2	0.66				
p-S6rp (0–3, linear)	206	1.2	0.75-2.1	0.41				
Histologic grade (3 vs 1–2)	204	2.7	1.4-5.2	0.004				
PgR (pos vs neg)	206	0.32	0.16-0.63	0.001				

^a^P-value for Cox-regression.

^b^Multivariable analysis adjusted for node positivity, tumor size, HER2, ER, Ki67 and menopausal status.

^c^Multivariable analysis adjusted for age, tumor size, HER2, ER and Ki67.

**Table 3:** **Prognostic value of IGF1R intensity in ER-negative and ER-positive patients in Cohort I (a) and II (b), respectively**

a)									
Cohort I		DDFS univariable				DDFS multivariable ^a^			
		N	HR	95% CI	p-value	N	HR	95% CI	p-value
Cytoplasm	All patients	264	0.70	0.52-0.94	0.016	220	0.80	0.57-1.1	0.18
	ER+	174	1.2	0.76-2.0	0.40	152	1.21	0.72-2.0	0.46
	ER-	80	0.62	0.40-0.96	0.033	68	0.49	0.29-0.82	0.007
Membrane	All patients	264	0.58	0.39-0.86	0.007	220	0.71	0.46-1.1	0.13
	ER+	174	0.89	0.54-1.5	0.63	152	0.91	0.54-1.5	0.72
	ER-	80	0.44	0.20-0.97	0.041	68	0.32	0.13-0.79	0.014
**b)**									
**Cohort II**		**DDFS univariable**				**DDFS multivariable** ^**b**^			
		**N**	**HR**	**95% CI**	**p-value**	**N**	**HR**	**95% CI**	**p-value**
Cytoplasm	All patients	206	0.87	0.52-1.5	0.61	179	1.0	0.59-1.8	0.91
	ER+	139	1.1	0.48-2.5	0.83	122	1.2	0.53-2.9	0.62
	ER-	67	0.81	0.42-1.56	0.53	57	0.71	0.32-1.6	0.39
Membrane	All patients	206	0.87	0.53-1.4	0.57	179	1.03	0.57-1.9	0.92
	ER+	139	1.3	0.64-2.5	0.51	122	1.2	0.59-2.5	0.60
	ER-	67	0.72	0.34-1.5	0.37	57	0.84	0.31-2.3	0.74

^a^Multivariable Cox-regression for IGF1R intensity adjusted for node positivity, tumor size, HER2, Ki67, menopausal status and, among all patients, ER status.

^b^Multivariable Cox-regression for IGF1R intensity adjusted for age, tumor size, HER2, Ki67 and, among all patients, ER status.

When stratifying for ER status (Table 3a, Figure 4b) the prognostic effect was found in the ER-negative (HR = 0.62, 95% CI = 0.40 – 0.96, p = 0.033) but not in the ER-positive group (HR = 1.2, 95% CI = 0.76 – 2.0, p = 0.40). The difference between ER-negative and ER-positive patients was confirmed in interaction analysis (HR = 2.0 for IGF1R in ER-positive compared to ER-negative patients, p = 0.038). Thus, there was moderate evidence that the influence of IGF1R on prognosis was stronger in the ER-negative group. The interaction remained after multivariable adjustment for tumor size, node status, HER2, Ki67, and menopausal status (p = 0.054 in interaction analyses). Multivariable analyses after stratification based on ER status also showed that the prognostic value of IGF1R only remained in the ER-negative subgroup (Table 3a). Analyzing only postmenopausal (N = 209) or only node-positive patients (N = 178) in Cohort I increased the effect of IGF1R intensity on survival by lowering HR to 0.58 (95% CI = 0.39 – 0.85, p = 0.006) and 0.61 (HR = 0.61, 95% CI = 0.43 – 0.87, p = 0.006), respectively. However, the difference between pre- and post-menopausal as well as between node-positive and node-negative patients could not be established in interaction analyses (p = 0.37 and p = 0.18, respectively). p-mTOR and p-S6rp expression showed no significant relation to survival in neither Kaplan-Meier analyses (see Additional file 2) nor Cox-regression analyses (Table 2a).

In Cohort II where only 9 patients had received endocrine treatment, all women were premenopausal and node-negative. No significant prognostic value could be found for IGF1R intensity using neither Kaplan-Meier analyses nor Cox-regression (Table 2b and Figure 4a). Excluding the 28 patients in Cohort II that had received adjuvant systemic therapy did not give divergent results (data not shown). Of the 206 tumors that could be evaluated for IGF1R, 67 samples were ER-negative and 139 were ER-positive but ER-stratification did not provide any prognostic information for the experimental markers (Table 3b and Figure 4c). No prognostic value could be found for p-mTOR and p-S6rp intensity in Cohort II (Additional file 2; Table 2b), but high p-mTOR fraction gave moderate evidence for decreased survival (HR = 0.98, 95% CI = 0.97 – 1.0, p = 0.035).

## Discussion

Our hypothesis at initiation of the study was that over-activation of the IGF1R pathway could lead to tamoxifen resistance through for example ligand independent activation of ER [12, 17, 36]. This is in line with biological reasoning based on the growth promoting and anti-apoptotic function of IGF1R. However, our results showed that patients with *negative* IGF1R expression had significantly worse prognosis and that phosphorylation of downstream markers mTOR and S6rp was not associated to prognosis. Taken together these results suggest that the hypothesis can be rejected.

In more detail, we could show that IGF1R negativity was associated with shorter distant disease-free survival (DDFS) in a cohort of postmenopausal women with stage II breast carcinoma. Other studies have also found results indicating an advantageous effect of high IGF1R or an association between tamoxifen resistance and low IGF1R [18–21]. A tamoxifen resistant cell line was found to have decreased levels of IGF1R, and treatment with IGF1R inhibiting antibodies had no effect on proliferation and cell growth [37]. Cell line experiments even suggest that high IGF1R expression could be used as a marker for endocrine treatment sensitivity [38]. Our results can be interpreted as indicative of the same conclusion since shorter DDFS for patients with low IGF1R expression was found only in the tamoxifen treated Cohort I. In Cohort II, consisting of premenopausal women without tamoxifen treatment, no prognostic value of IGF1R expression could be found. But it has to be considered that only 5 patients in Cohort II (compared to 30 patients in Cohort I) were IGF1R-negative, which might hide a possible effect of IGF1R expression on survival. No association was found between the experimental markers with the exception of IGF1R expression in cytoplasm and membrane. This indicates that there was no specific activation of the pathway in these patients. However, the stability of phospho-epitopes has rightfully been questioned [39] and the risk that pre-analytic handling of the samples could affect this expression should be considered. In the present study, no information regarding treatment of individual samples is available but all samples have been routinely handled according to good laboratory practice in established pathology departments.

We found an association between high IGF1R and ER positivity in Cohort I. Comparison between St Gallen breast cancer subgroups [34] showed significantly higher expression of IGF1R in Luminal A and B-like subclasses compared to Triple-negative and HER2-positive classes in Cohort I. This clearly demonstrates strong positive association between IGF1R and ER expression. Other studies have also found that IGF1R correlates with “good” prognostic factors such as high ER expression [17] and it has been suggested that IGF1R expression, in accordance with ER, reflects a well differentiated tumor [19, 20]. Both *in vitro* and *in vivo* studies have shown that mammary tumors induced by IGF1R have weak metastatic capacity and that lowered expression of IGF1R is essential for increased cell motility [40, 41]. When analyzing only node-positive patients in Cohort I we found the prognostic value of IGF1R expression to be higher (however not significant in interaction analysis) compared to its value in the whole cohort, suggesting a possible association between lymph-node spread and the prognostic value of IGF1R negativity. In summary, there are previous studies suggesting that high expression of IGF1R is indicative of a well differentiated tumor with weak metastatic capacity. Our results support this notion although further studies have to be performed for conclusive evidence.

Interestingly, we only found a prognostic value of IGF1R expression in ER-negative patients in Cohort I. A previous study has suggested that ER-negative patients are indeed more sensitive to growth promoting signals from IGF1 since they have constant expression of IGF1R and their response to IGF1 is thus not under estrogen control [7]. When analyzing IGF1R expression only in the postmenopausal women of Cohort I, the effect on survival was higher. Together with the rest of the results this suggests that the prognostic signal in this study comes mainly from ER-negative, postmenopausal and node-positive patients and that IGF1R negativity is associated with worse survival mainly in this subgroup of Cohort I. Higher expression of IGF1R might not reflect increased activation of the complete system and we found no evidence of pathway activation in neither mTOR nor S6rp downstream of highly expressed IGF1R. Co-activation of several receptor tyrosine kinases (RTKs) has been found in cell lines and this could possibly explain why blocking a single RTK often has marginal effect [42]. Downstream signaling from IGF1R can be transmitted either by the mTOR or by the MAPK pathway, but since the two pathways converge before activating S6K [10] it is unlikely that downstream activation in any of the pathways was correlated with IGF1R expression. Contradictory to several other studies [22–24], we found no evidence of a prognostic value of either p-mTOR or p-S6rp intensity. Only the fraction of expressed p-mTOR was associated with decreased survival in Cohort II.

## Conclusions

We found that IGF1R expression could be positively associated with hormone receptor expression in one cohort of postmenopausal, tamoxifen treated women. We also found that lack of IGF1R expression was indicative of inferior survival in the same cohort, mainly in ER-negative patients. The underlying mechanisms for these results need further investigation in order to elucidate the role of IGF1R in the development of resistance against endocrine therapy in breast cancer.

## Electronic supplementary material

Additional file 1: **Expression of experimental markers in relation to tumor and patient characteristics.** **a)** IGF1R membrane expression, **b)** p-mTOR expression and **c)** p-S6rp expression. (PDF 480 KB)

Additional file 2: **Kaplan-Meier kurves for distant disease-free survival (DDFS) in the two cohorts.** **a)** IGF1R membrane expression **b)** p-mTOR expression and **c)** p-S6rp expression. (PDF 162 KB)
