# Supplementary material for: Association between insulin-like growth factor-1 receptor (IGF1R) negativity and poor prognosis in a cohort of women with primary breast cancer
Source: BMC Cancer. 2014 Nov 3;14:794. doi: 10.1186/1471-2407-14-794 (PMC4232733; doi:10.1186/1471-2407-14-794)
Supplement: Supplementary file 1 — Additional file 1: Expression of experimental markers in relation to tumor and patient characteristics. a) IGF1R membrane expression, b) p-mTOR expression and c) p-S6rp expression. (PDF 480 KB) [file 12885_2014_4985_MOESM1_ESM.pdf]

Additional file 1 (pdf). Expression of experimental markers in relation to tumor and patient characteristics.

a) IGF1R membrane	Cohort I	% of patients with different expression levels					Cohort II	% of patients with different expression levels				
	N	neg	weak	moderate	strong	p-value	N	neg	weak	moderate	strong	p-value
**Total**	264	35	53	8	3		206	28	59	10	3	
**Age**												
(continuous)	264	63[a]	63[a]	64[a]	60[a]	1.0[b]	206	46[a]	47[a]	47[a]	48[a]	0.19[b]
**Menopausal status**												
pre	55	33	60	4	4	0.97[c]	n/a					
post	209	36	52	10	3							
**Tumor size**												
0 – 20 mm	78	40	51	9	0	0.24[c]	156	27	60	11	2	0.87[c]
>20 mm	186	33	54	8	4		50	30	56	8	6	
**Node status**												
N0	86	34	56	8	2	0.88[c]	n/a					
N+	178	36	52	8	3							
**NHG**												
1 – 2	186	30	58	9	3	0.021[c]	138	23	62	12	4	0.023[c]
3	75	48	41	7	4		66	38	53	8	2	
missing	3						2					
**ER**												
positive	174	25	59	11	5	<0.001[c]	139	23	61	13	3	0.016[c]
negative	80	60	38	3	0		67	37	55	4	3	
missing	10						0					
**PgR**												

positive	133	24	62	11	2	<0.001[c]	149	24	62	11	3	0.14[c]
negative	121	49	41	6	4		57	37	51	9	4	
missing	10						0					
**Ki67**												
low	162	31	57	8	4	0.25[c]	125	27	61	10	2	0.75[c]
high	99	39	49	9	2		61	30	52	13	5	
missing	3						20					
**HER2**												
negative	199	32	56	9	4	0.026[c]	171	25	60	12	3	0.066[c]
positive	33	52	42	3	3		22	41	55	5	0	
missing	32						13					
**St Gallen subgroups[d]**												
Luminal A-like	72	25	61	13	1	<0.001[e]	92	20	64	13	3	0.07[e]
Luminal B-like	80	23	59	10	9		32	25	56	16	3	
Triple-negative	42	64	31	5	0		32	41	47	9	3	
HER2+ (non-luminal)	18	72	28	0	0		8	50	50	0	0	
missing	52						42					

Abbreviations: ER = estrogen receptor, PgR = progesterone receptor, HER2 = human epidermal growth factor receptor 2, NHG = Histological grade according to Elston and Ellis (42), n/a = not applicable

[a] Median age in the different groups

[b] Spearman's rank-correlation

[c] Mann-Whitney test

[d] See (34) for complete definition of St Gallen subgroups

[e] Kruskal-Wallis test

b) p-mTOR	Cohort I	% of patients with different expression levels					Cohort II	% of patients with different expression levels				
	N	neg	weak	moderate	strong	p-value	N	neg	weak	moderate	strong	p-value
**Total**	264	35	5	17	44		206	23	3	16	58	
**Age**												
(continuous)	264	61[a]	62[a]	64[a]	62[a]	0.76[b]	206	46[a]	43[a]	47[a]	48[a]	0.026[b]
**Menopausal status**												
pre	55	38	0	9	53	0.52[c]	n/a					
post	209	34	6	19	42							
**Tumor size**												
0 – 20 mm	78	31	5	18	46	0.60[c]	50	21	3	16	60	0.10[c]
>20 mm	186	37	4	16	43		156	30	6	14	50	
**Node status**												
N0	86	38	6	21	35	0.29[c]	n/a					
N+	178	33	4	15	48							
**NHG**												
1 – 2	186	32	4	17	47	0.21[c]	139	13	2	19	66	0.63[c]
3	75	41	7	13	39		65	46	6	9	38	
missing	3						2					
**ER**												
positive	174	27	3	16	55	0.068[c]	140	13	2	16	69	0.014[c]
negative	80	53	6	16	25		66	45	6	15	33	
missing	10						0					
**PgR**												
positive	133	26	4	14	57	0.84[c]	150	15	2	15	68	0.23[c]

negative	121	45	4	18	32		56	46	7	16	30	
missing	10						0					
**Ki67**												
low	162	31	5	17	47	<0.001[c]	126	13	2	16	69	0.010[c]
high	99	41	4	14	40		61	44	7	13	36	
missing	3						19					
**HER2**												
negative	199	36	5	17	43	0.44[c]	171	24	4	15	57	0.13[c]
positive	33	24	3	18	55		22	9	0	18	73	
missing	32						13					
**St Gallen subgroups**[d]												
Luminal A-like	72	28	4	13	56	<0.001[e]	93	11	2	16	71	<0.001[e]
Luminal B-like	80	25	1	18	56		32	13	0	16	72	
Triple-negative	42	67	5	14	14		31	65	13	10	13	
HER2+ (non-luminal)	18	39	6	22	33		8	25	0	13	63	
missing	52						42					

Abbreviations: ER = estrogen receptor, PgR = progesterone receptor, HER2 = human epidermal growth factor receptor 2, NHG = Histological grade according to Elston and Ellis (42), n/a = not applicable

[a] Median age in the different groups

[b] Spearman's rank-correlation

[c] Mann-Whitney test

[d] See (34) for complete definition of St Gallen subgroups

[e] Kruskal-Wallis test

c) p-S6rp	Cohort I	% of patients with different expression levels					Cohort II	% of patients with different expression levels				
	**N**	**neg**	**weak**	**moderate**	**strong**	**p-value**	**N**	**neg**	**weak**	**moderate**	**strong**	**p-value**
**Total**	264	9	12	37	42		207	6	1	19	73	
**Age**												
(continuous)	264	61[a]	64[a]	63[a]	61[a]	0.40[b]	207	47[a]	51[a]	47[a]	47[a]	0.16[b]
**Menopausal status**												
pre	55	38	0	9	53	0.51[c]	n/a					
post	209	34	6	19	42							
**Tumor size**												
0 – 20 mm	78	3	9	50	38	0.47[c]	157	5	2	17	76	0.73[c]
>20 mm	186	11	13	31	44		50	8	0	28	64	
**Node status**												
N0	86	9	15	24	51	0.098[c]	n/a					
N+	178	8	11	43	38							
**NHG**												
1 – 2	186	11	12	37	40	0.14[c]	139	8	1	18	73	<0.001[c]
3	75	3	13	39	45		66	2	2	23	74	
missing	3						2					
**ER**												
positive	174	10	12	38	40	<0.001[c]	140	9	1	21	69	<0.001[c]
negative	80	5	11	33	51		67	0	1	15	84	
missing	10						0					
**PgR**												
positive	133	11	8	38	43	<0.001[c]	150	7	2	20	71	<0.001[c]

negative	121	7	16	35	43		57	4	0	18	79	
missing	10						0					
**Ki67**												
low	162	11	16	41	32	0.14[c]	126	7	1	22	70	<0.001[c]
high	99	4	6	29	61		61	2	0	11	87	
missing	3						19					
**HER2**												
negative	199	9	11	38	43	0.15[c]	172	7	1	20	72	0.10[c]
positive	33	9	21	30	39		22	0	0	14	86	
missing	32						13					
**St Gallen subgroups[d]**												
Luminal A-like	72	14	10	39	38	0.20[e]	93	9	0	26	66	0.20[e]
Luminal B-like	80	6	15	39	40		32	9	0	6	84	
Triple-negative	42	5	10	29	57		32	0	0	22	78	
HER2+ (non-luminal)	18	11	22	22	44		8	0	0	25	75	
missing	52						42					

Abbreviations: ER = estrogen receptor, PgR = progesterone receptor, HER2 = human epidermal growth factor receptor 2, NHG = Histological grade according to Elston and Ellis (42), n/a = not applicable

[a] Median age in the different groups

[b] Spearman's rank-correlation

[c] Mann-Whitney test

[d] See (34) for complete definition of St Gallen subgroups

[e] Kruskal-Wallis test
