# Supplementary figures and images for: Association between insulin-like growth factor-1 receptor (IGF1R) negativity and poor prognosis in a cohort of women with primary breast cancer
Source: BMC Cancer. 2014 Nov 3;14:794. doi: 10.1186/1471-2407-14-794 (PMC4232733; doi:10.1186/1471-2407-14-794)

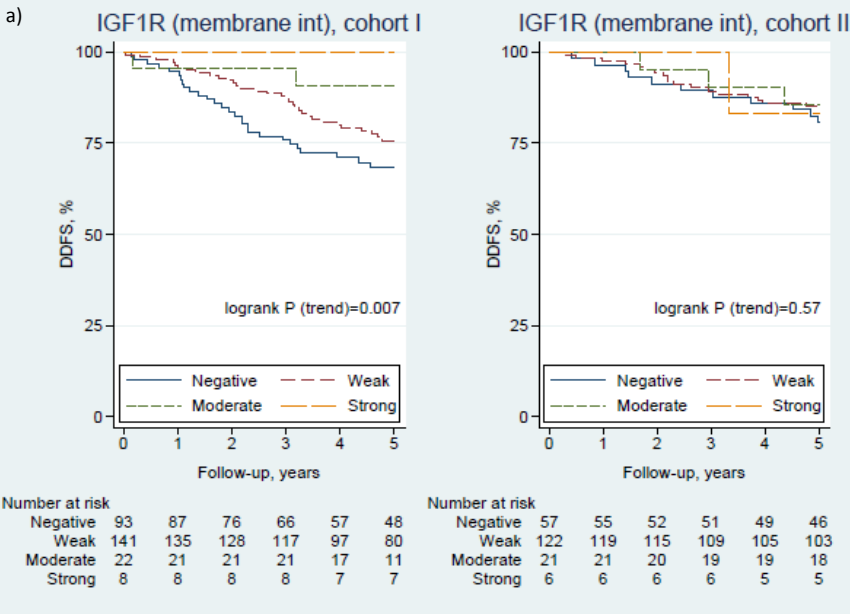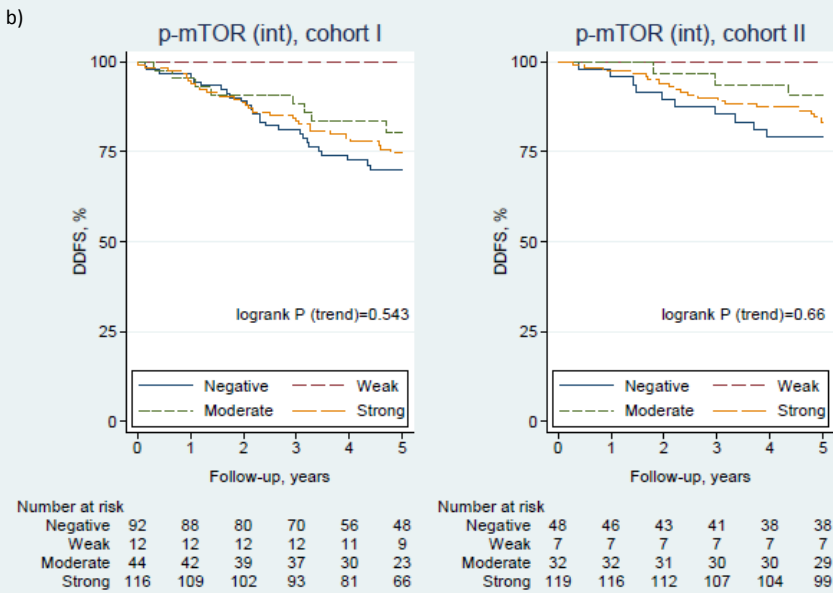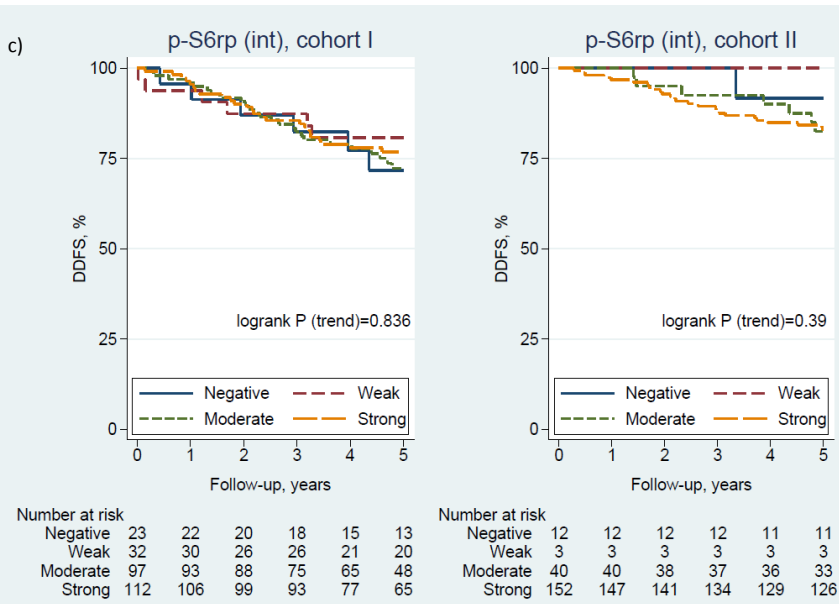

Supplement: Supplementary file 2 — Additional file 2: Kaplan-Meier kurves for distant disease-free survival (DDFS) in the two cohorts. a) IGF1R membrane expression b) p-mTOR expression and c) p-S6rp expression. (PDF 162 KB) [file 12885_2014_4985_MOESM2_ESM.pdf]
